# Factor structure of the HIV-SM LMIC self-management questionnaire for people living with HIV in low- and middle-income countries

**DOI:** 10.1186/s12981-024-00676-7

**Published:** 2024-12-21

**Authors:** Tegene Legese Dadi, Girmay Medhin, Mark Spigt

**Affiliations:** 1 College of Medicine & Health Science, School of Public Health, Hawassa University, Hawassa, Ethiopia; 2 School CAPHRI, Care and Public Health Research Institute, Maastricht University, Maastricht, The Netherlands; 3 Aklilu Lemma Institute of Pathobiology, Addis Ababa University, Addis Ababa, Ethiopia; 4 MERQ Consultancy PLC, Addis Ababa, Ethiopia; 5 General Practice Research Unit, Department of Community Medicine, The Arctic University of Tromsø, Tromsø, Norway

**Keywords:** HIV-SM LMIC questionnaire, Self-management questionnaire, HIV management, Developing countries

## Abstract

**Introduction:**

Despite the need for reliable questionnaires to monitor self-management in chronic disease patients, such tools are lacking in developing countries. This study aims to pilot and assess the construct validity of the HIV-SM LMIC questionnaire.

**Method:**

The validation of the HIV-SM LMIC questionnaire involved two cross-sectional studies in Ethiopia. The first round, for exploratory factor analysis (EFA), included 261 patients, while the second round, for confirmatory factor analysis (CFA), included 300 patients. Data was collected using the Kobo Collect electronic data entry template.

**Result:**

The sample adequacy test showed a good value of 0.82. In the first round, 6 of the 32 items were not loaded, forming three factors in the EFA. Four of these items were dropped, but two (PSMB2 and PSMB12) were retained for their content. In the second round, CFA on the remaining 28 items led to dropping 8 more items due to conceptual overlap, resulting in a 20-item questionnaire. The final items were structured into three dimensions: awareness and well-being (4 items), self-regulation (6 items), and self-management practices (10 items).

**Conclusion:**

The study refined the original 32-item HIV-SM LMIC questionnaire to a validated 20-item, three-dimensional tool with an acceptable goodness of fit. The authors recommend further cross-cultural and predictive validation and adaptation for newly diagnosed HIV patients, those with poor treatment outcomes.

**Supplementary Information:**

The online version contains supplementary material available at 10.1186/s12981-024-00676-7.

## Introduction

The introduction of antiretroviral therapy (ART) and substantial investments in care and support of HIV patients have saved lives and transformed the previously life-threatening HIV disease into manageable chronic diseases [1, 2]. According to UNAIDS, HIV treatment has reduced AIDS-related deaths by 69%, equivalent to 20.8 million deaths averted since 2004. In addition, 29.8 million of the 39 million people living with HIV worldwide are receiving life-saving treatment [3]. Despite these positive improvements, the disease remains a major challenge for health systems and patients, with 2.5 million new HIV infections and 2 million deaths each year [3–5].

People living with HIV face multidimensional problems, including economic, social and psychological problems [6, 7]. HIV-related discrimination has contributed to the loss of employment for more than 50% of people living with HIV in some countries, and up to 21% report being denied health care [7]. These problems lead to poor quality of life, social alienation and mental health problems [8–11]. A study in Africa showed that HIV patients exposed to stigma were twice as likely to have poor health-related quality of life [8]. In addition to patients, families also face economic strain and poverty, and experience increased caregiving responsibilities as the disease progresses [12–14]. Studies show that HIV reduces household income by 10–14% [13, 15], increases the burden on the health care system [16], increases non-communicable diseases by up to 10% [17], and increases HIV treatment failure by up to 24% [18, 19]. In developing countries the growing number of HIV patients, coupled with the aging HIV population and the rising risk of multi-comorbidities, is putting additional strain on the healthcare system [2, 20]. There is also an increase in HIV drug resistance, with 10–25% pretreatment drug resistance to NVP or EFV [21] and 3–29% acquired drug resistance [22].

To address the challenges mentioned, it is essential to rethink the current service delivery model, shifting from provider-centered to patient-centered care. Successful treatment relies heavily on patient engagement for two main reasons: (1) patients have a deeper understanding of their condition [23, 24]; and (2) better outcomes occur when patients are actively involved in managing their care, as they make daily decisions affecting their health [23, 25]. Empowering patients through self-management encourages responsibility for their health, leading to improved outcomes, higher quality care, reduced burden on the healthcare system, better coping with stigma and discrimination, and fewer hospitalizations [26–30]. For example, in developing countries, the demand for self-management is increasing due to the rising HIV caseload and the chronic nature of the disease [16, 31]. Overall, self-management interventions offer a holistic approach, addressing individual, social, and health system factors and tailoring care to the patient’s needs [28, 32–34].

While implementing self-management practices in everyday healthcare, it also becomes essential to assess self-management. For chronic disease patients, it is crucial to use a structured questionnaire to monitor and measure regular self-management practices that can potentially affect treatment outcomes [35–37]. Several self-management assessment tools exist for evaluating self-management practices among HIV patients. Yet, many of these tools have limitations and lack comprehensiveness. Some are tailored to specific populations, such as a tool developed by Talitha et al. [38], which focuses on adolescents, and one by Wabel et al., which targets women in developed countries [39]. Other tools focus on specific health-related issues, such as the tool by Kenneth et al., which assesses perceived medical conditions [40], or the tool by Mallory et al., which emphasizes adherence [41]. However, there is a lack of reliable, comprehensive, and valid questionnaires that accurately assess self-management in the context of developing countries [28, 42–44].

The aim of this research is to extend the groundwork laid by previous research on the development of HIV-SM LMIC questionnaire, which involved item generation and testing of its face and content validity for the context of developing countries [45]. The primary objective of this study is to pilot and assess the construct validity of the HIV-SM LMIC questionnaire.

## Methods

### Study design

The validation process of the HIV-SM LMIC questionnaire employed an observational research method, specifically a cross-sectional study design, to collect data from selected HIV patients who visited health facilities for healthcare services in Ethiopia. Study participants were selected from Hawassa University Comprehensive Specialized Hospital (HUCSH), Adare General Hospital, and Millennium Health Center in two rounds study (for exploratory factor analysis and confirmatory factor analysis).

### Study setting

Ethiopia is the second most populous country of Africa and ranks 12th in the world with a population of more than 105 million [46]. The country experiences rapid population growth (2.6%) has a young age structure, and a high dependency ratio, with significant rural-urban differences [47]. Ethiopia’s healthcare system is organized into three tiers: primary, secondary, and tertiary levels of care. Primary health care units provide essential services, general hospitals offer secondary care, and specialized hospitals deliver tertiary care [48]. The study was conducted at HUCSH, a tertiary-level facility; Adare General Hospital, a secondary-level facility; and Millennium Health Center, a primary-level facility. These facilities are selected because of various reasons including diversity of HIV patients from where comes from various regions; and levels of the health facility which will give us the opportunity to acquire different contexts.

HUCSH serves approximately 5 million people from the southern regions of Ethiopia, predominantly drawing users from Sidama, Oromia, Southwest Ethiopia, and Somali regions. The hospital, equipped with over 500 beds, has registered 7,967 HIV patients since the initiation of ART, with 2,760 active adult HIV patients (1,726 female and 1,072 male) during the current study. Adare General Hospital serves a catchment population of approximately 1.3 million, primarily from the Sidama region and partly from the West Arsi zone (Oromia region). With 120 beds, the hospital has registered 3,205 HIV patients since the inception of ART, among whom 2,434 (1,559 females and 875 males) are active adult HIV patients during the current study. Millennium Health Center provides health services to around 83,215 individuals from two sub-cities and five kebeles of Hawassa city. Since its establishment, the health center has registered 892 ART patients, with 601 active adult HIV patients (401 female and 200 male) during the current study.

### Context of the study

This study is part of a series of studies aimed at developing a comprehensive self-management questionnaire tailored for HIV patients in developing countries. As part of this undertaking, three research outputs have already been produced which was the basis for the current study; two published papers [23, 24], and the development and validation (face and content validation) of HIV-SM LMIC questionnaire, currently under review [45]. The present study delves into evaluating the construct validity of the 32-item HIV-SM LMIC questionnaire through two stages: a first-round study to explore its factor structure using exploratory factor analysis, followed by confirmatory factor analysis.

### Sample size and recruitment of study participants

Both the first-round study and confirmatory factor analysis were conducted among adult HIV patients, specifically those above 18 years of age, who were in follow-up appointments at the health facilities. Newly diagnosed HIV patients were not included in both rounds of the assessment. The sample size for both first-round study and confirmatory factor analysis was determined based on recommendations provided by various authors. Different authors suggest minimum sample sizes ranging from 100 to 1,000 for testing questionnaire reliability and validity in exploratory and confirmatory factor analysis [49, 50]. However, most agree that a sample size between 200 and 300 is sufficient [50]. Following these recommendations, the first-round study for exploratory factor analysis included a sample size of 260 patients, and 300 patients for the confirmatory factor analysis. Patients were enrolled in the study during their visits to the health facility, after completing their service delivery. Before starting the interview, patients were informed about the study and consented to participate. Enrolment was conducted consecutively until the sample size was reached.

### The process of data collection

There were two rounds of data collection and one round of discussion with the data collectors. The first round of data collection was to collect data that could be used for exploratory factor analysis (EFA), and the second round was to collect data that could be used for confirmatory factor analysis (CFA). Before commencing data collection, the data collectors were trained on the questionnaires and began data collection once they were thoroughly familiar with them. The discussion with data collectors was conducted after the first round of data collection. Notes from the discussion were recorded item by item in a notebook.

Both rounds of quantitative data were collected through face-to-face interviews by trained data collectors, who were healthcare providers within the ART clinics of the study health facilities. Data collection tools were prepared using the Kobo Collect electronic data entry template. Data were collected using smart mobile phones, and the data collectors sent the data to the server daily. A discussion with the data collectors was held after the completion of the pilot data or first-round of data collection. The main focus of the discussion was on the items and patients’ responses and with the aim of identifying areas for improvement for the second round of data collection.

### Quality assurance of the data

The data quality assurance (DQA) was ensured through various measures including a comprehensive training for data collectors to ensure their understanding of the items, along with pre-testing of the survey tools. Field supervisor provided supervision to all data collectors to check completeness and quality. The researcher (TLD) closely monitored the data collection process daily to ensure data quality using data quality queries. Any records identified with data quality issues during data collection were discussed with data collectors for corrections. Daily feedback sessions were organized with each data collector through phone calls and face-to-face meetings.

### Data management and analysis

Data was exported to Stata version 16 for management and analysis. Frequency distribution of variables was produced to identify possible errors and outliers in the responses. Descriptive results were presented using tables. Exploratory factor analysis (EFA) was conducted using data collected in the first round to examine preliminary dimensions of the questionnaire and to screen items that potentially could contribute to the confirmatory factor analysis (CFA). Sample adequacy was tested for overall and for individual items. A multivariate normality test was performed to assess the normal distribution of the items. The results informed the selection of exploratory factor analysis rotation methods. The number of factors selected was based on the dimensions of the Individual and Family Self-Management Theory (IFSMT) model. Based on the results of the EFA, a discussion was held with the data collectors to gather their reflections on their interview experiences and patients’ responses to each item. Discussion points included how well the items captured the correct responses from patients, issues identified with items or their response categories or scales, the importance of the items, and types of responses. Using the data collected in the second round, confirmatory factor analysis (CFA) was conducted using maximum likelihood estimation.

## Results

A total of 561 HIV patients (261 in the first round and 300 in second round study) were interviewed for the validation and investigation of factor structure of HIV-SM LMIC questionnaire.

### Characteristics of study participants

#### Socio-demographic characteristics

In both rounds of data collection, most patients were in the age range of 35–39 years, with the fewest patients coming from the 18–24 years age range. More than two-thirds of the respondents were female in both rounds, and one-third had a minimum of secondary school level of education. Around one-third were merchants or self-employed, nearly half of patients are below the extreme poverty line. More than 40% of respondents in the first round and 30% in the second round had to travel for more than 10 km to visit the health facility. Approximately 11% in the first round and 7% of HIV patients in the second round had to travel more than 100 km to reach the health facility. More than 20% in the first round and over 10% in the second round spend more than 5% of their monthly income on travel to the health facility (Table 1).

**Table 1:** Socio-demographic characteristics of HIV patients that participated in the study

Variable	Categories	First-round study # (%)	Second round study # (%)
Name of the health facility	HU CMHS hospital	174 (66.67)	136 (45.33)
	Adare Hospital	85 (32.57)	99 (33.00)
	Millenium Health center	2 (0.77)	65 (21.67)
Age of respondent	18–29 years	38 (15.14)	38 (12.92)
	30–39 years	92 (36.65)	120 (40.82)
	40–49 years	90 (35.85)	100 (34.02)
	50 + years	31 (12.35)	36 (12.24)
Sex of the patient	Female	180 (71.71)	204 (68.00)
	Male	71 (28.29)	96 (32.00)
Marital status	Single	27 (10.34)	21 (7.00)
	Currently Married	139 (53.26)	170 (56.67)
	Widowed	38 (14.56)	54 (18.00)
	Divorce	57 (21.84)	55 (18.33)
Level of education of the patient	No formal education	22 (8.76)	30 (10.20)
	Primary education (1–8 grades)	77 (30.68)	96 (32.65)
	Secondary education (9–12 grades)	93 (37.05)	95 (32.32)
	Level I-V	36 (14.34)	45 (15.30)
	Degree and above	23 (9.16)	28 (9.52)
Occupation	Housewife	55 (21.91)	52 (17.69)
	Merchant/Self-employed	78 (31.08)	105 (35.71)
	Student	12 (4.78)	5 (1.70)
	Government/private employee	62 (24.70)	75 (25.51)
	Daily laborer	33 (13.15)	46 (15.65)
	Others	11 (4.38)	11 (3.74)
Extreme Poverty line	Below extreme poverty line	158 (60.54)	148 (49.33)
	Above extreme poverty line	103 (39.46)	152 (50.67)
Travel distance in KM to come to the health facility	Less than 10 KMs	156 (59.77)	208 (69.33)
	11–25 KMs	37 (14.18)	34 (11.33)
	26–50 KMs	27 (10.34)	23 (7.67)
	51–100 KMs	13 (4.98)	14 (4.67)
	101 + KMs	28 (10.73)	21 (7.00)
% of monthly income utilized for transport	<=1% of income	89 (36.93)	144 (49.32)
	1.1-5% of income	103 (42.74)	119 (40.75)
	5–10% of income	18 (7.47)	16 (5.48)
	> 10% of income	31 (12.86)	13 (4.45)

HU CMHS: Hawassa University College of Medicine and Health Science; KM: Kilometer

#### Clinical characteristics of study participants

Table 2 describes the clinical characteristics of the study participants enrolled in two rounds. More than one in seven of HIV patients included in both two rounds of study did not disclose their HIV status to their family or peers. The majority in the first round (28.3%) were on ART regimens for 10–15 years, whereas most of the respondents included in the second round were on ART for 5–10 years. Over three-fourths of HIV patients in both rounds had changed their ART regimen at least once. Around 7% of HIV patients were on second-line ART regimens. Most patients in both rounds of data collection had undetectable viral loads, which is below 50 copies of the virus per ml. More than 12% of HIV patients had at least one non-communicable disease. Additionally, more than one-fifth of HIV patients experienced at least a mild form of psychological distress.

**Table 2:** Clinical characteristics of HIV patients that participated in the study

Variable	Categories	First round study # (%)	Second round study # (%)
Disclosed HIV status	No	40 (15.33)	41 (13.67)
	Yes	221 (84.67)	259 (86.33)
Months on ART	Less than 1 year	17 (6.77)	7 (2.38)
	Between 1–5 years	53 (21.12)	66 (22.44)
	Between 5–10 years	64 (25.5)	92 (31.29)
	Between 10–15 years	71 (28.29)	84 (28.57)
	More than 15 years	46 (18.33)	45 (15.31)
Baseline viral load	Below 50 copies	236 (94.02)	268 (91.16)
	51–499 copies	6 (2.39)	9 (3.06)
	500 + copies	9 (3.59)	17 (5.78)
Frequency of regimen change	No change	62 (24.7)	78 (26.53)
	One times	144 (57.37)	155 (52.72)
	Two times	25 (9.96)	50 (17.01)
	3 + times	20 (7.97)	11 (3.74)
Current ART regimen type	First line ART regimen	233 (92.83)	272 (93.16)
	Second line ART regimen	18(7.18)	20(6.80)
Last two viral load: preceding	Below 50 copies	236 (94.02)	273 (92.86)
	51–499 copies	7 (2.79)	9 (3.06)
	500 + copies	8 (3.19)	12 (4.08)
Last two viral load: recent	Below 50 copies	246 (98.01)	287 (97.62)
	51–499 copies	3 (1.20)	7 (2.38)
	500 + copies	2 (0.80)	
Presence of NCD	No	220 (87.65)	255 (86.73)
	Yes	31 (12.35)	39 (13.27)
Psychological distress	None	204 (78.16)	221 (73.67)
	Mild	52 (19.92)	67 (22.33)
	Moderate	1 (0.38)	9 (3.00)
	Severe	4 (1.53)	3 (1.00)

ART: Antiretroviral Therapy; NCD: Noncommunicable disease

### Validation of items of HIV-SM LMIC questionnaire

**Fig. 1:**
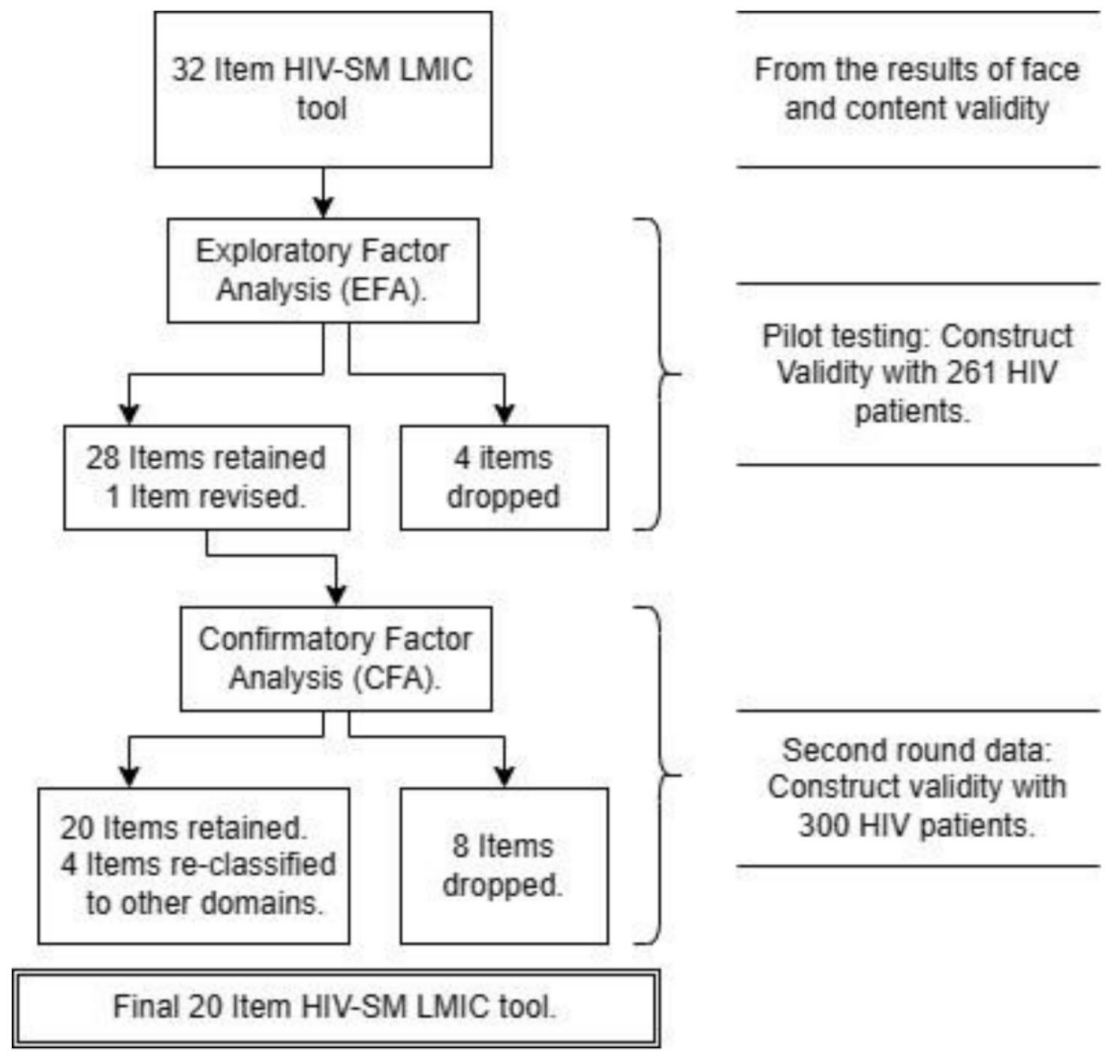
HIV-SM LMIC questionnaire validation process

Figure 1 illustrates the flow of the validation process. During the first-round study with exploratory factor analysis, 4 items were dropped, and one item was revised. In the CFA, 4 items were reclassified based on the EFA results, and 8 items were dropped, resulting in a final 20-item HIV-SM LMIC questionnaire. The following two subsections describe the results from EFA and CFA.

### Exploratory factor analysis

**Fig. 2:**
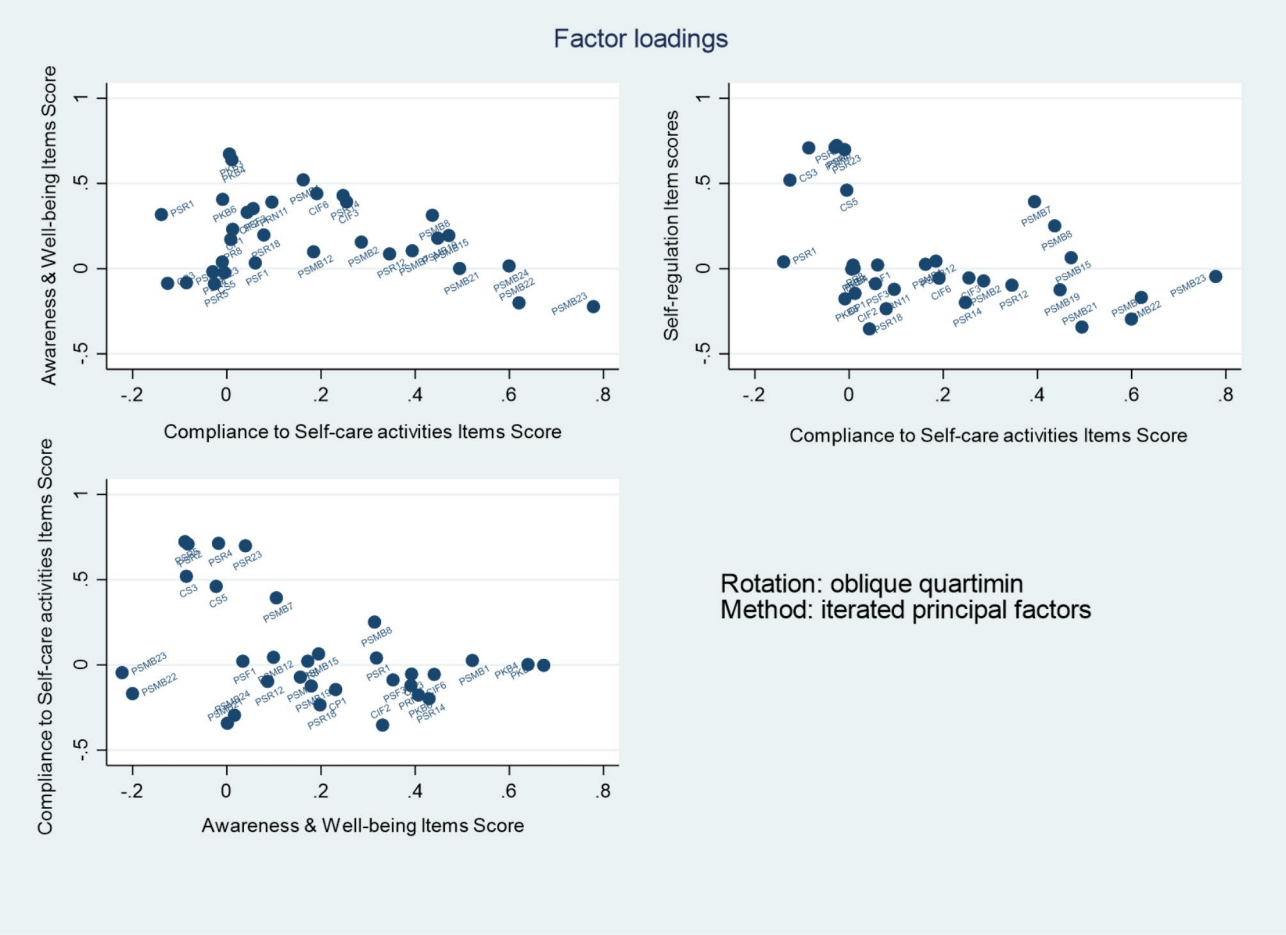
Rotated factor loading of items

As Table 3 showed that there is no significant difference in terms of the 32 items included in the first and second round patients. The initial construct validity of the 32-item HIV-SM LMIC questionnaire (6 items in contextual, 15 in process, and 11 in the proximal outcome dimension) was assessed using exploratory factor analysis (EFA) on first round study collected from 261 HIV patients. The number of factors to be extracted from the data was set to three, corresponding to these dimensions. The EFA was conducted using the iterated principal factors method, with quartimin oblique rotation to generate the results. We have chosen quartimin oblique rotation because the factors are correlated in which one of the factors affects the other factor. The factors that will be identified through EFA are not independent that is why we have used quartimin oblique rotation. The sample adequacy test showed a good overall value of 0.82. The rotated factor loading of items within the three dimensions are presented in Fig. 2.

**Table 3:** Exploratory factor analysis result - item loadings and grouping

Item code	Questions	% of PLWH with high** item score.		Loadings	IFSMT dimension
		1st round	2nd round		
**EFA new dimension 1**					
CIF3	Do you feel you have enough time to look after your health (e.g. taking medicines, rest…) despite your family responsibilities?	97.7	96.7	0.39	Context
CIF6	Do you agree that staying physically active (any movement at any time such as walking, cycling, and others for at least 2 days per week) is an important part of HIV care?	98.5	98.7	0.44	
PKB3	Do you believe that you have a clear understanding of HIV?	98.1	98.0	0.67	Process
PKB4	How often do you forget to take your medicine?	97.3	98.3	0.64	
PKB6	Do you agree that sufficient sleep is important for HIV care?	96.6	98.7	0.41	
PRN11	Do you trust the advice the health care providers give about your health and HIV?	99.6	99.0	0.39	
PSF3	Do you agree that you have a trustworthy person to turn to if you have problems?	91.2	90.0	0.35	
PSR1	Have you been encouraged to disclose your HIV status to your close family or peers?	88.9	89.7	0.32	
PSR14	Do you agree that you Integrate your treatment plan into your daily routine?	99.6	99.7	0.43	
PSMB1	To what extent do you use the knowledge of your current conditions to better manage HIV?	98.1	96.7	0.52	Proximal Outcome
**EFA new dimension 2**					
CS3*	Do you frequently feel alienated or isolated from others because of your HIV?	86.6	75.7	0.52	Context
CS5*	Do you frequently worry about disclosing your HIV to close family or peers?	78.9	80.7	0.46	
CIF2	Do you agree that reducing use of substances (drinking alcohol / using drugs / smoking khat or cigarettes) is critical for better HIV management?	97.3	99.0	-0.35	
PSR2*	How often do you skip or miss your medication when you are with people?	90.8	96.0	0.71	Process
PSR4*	How often do you forget to take your medicine?	89.7	89.3	0.71	
PSR5*	How often do you decide not to take your medicine?	91.6	88.7	0.72	
PSR23*	How often do you experience the feeling of hopelessness, depression, sadness or anxiety or related symptoms due to your illness?	89.3	94.7	0.70	
PSMB2	How strong are you when society excludes or isolates you from your social life due to your illness?	91.6	91.3	Not loaded	Proximal outcome
**EFA new dimension 3**					
PSR12	Do you agree that you should stick to your treatment plan even when side effects, which are not life threatening, bother you?	97.7	99.3	0.35	Process
PSMB7	To what extent are you self-disciplined enough to perform regular exercise?	78.2	85.7	0.39	Proximal Outcome
PSMB8	To what extent have you modified your diet (eating vegetables, fruits, or balanced diet what is available at home) to better manage HIV?	86.2	86.0	0.44	
PSMB12	To what extent are you able to stop unpleasant thoughts and frustration (e.g. anger, fear, sadness…) due to your illness?	82.8	87.7	Not loaded	
PSMB15	To what extent do you motivate yourself to set treatment goals to better manage HIV?	91.2	93.0	0.47	
PSMB19	To what extent do you strive to find effective solutions to problems related to your HIV disease management?	96.9	95.3	0.45	
PSMB21	To what extent are you implementing the HIV treatment strategy told to you by your health care providers?	96.6	98.3	0.49	
PSMB22	To what extent are you adhering to treatment medications uptake?	93.1	94.7	0.62	
PSMB23	To what extent are you adhering to medical follow-up schedules or appointments?	95.4	96.0	0.78	
PSMB24	To what extent do you take responsibility for your own health care?	97.3	97.0	0.60	
**Dropped items from further validation**					
CP1	Do you agree that the health facility offers the services you require most of the time, such as lab tests for viral load or CD4?	97.3	99.3	Not loaded	Context
PR8	Do you agree that the health care provider pays enough attention to your social or emotional problems (e.g. asking reasons for sadness or feelings…)?	91.6	91.7		Process
PSF1	Do you agree that it is important for you to attend support groups for your HIV management strategy?	87.7	96.0		
PSR18	How important is spirituality as a motivator to manage HIV?	100.0	99.7		

*Reverse scored items; EFA: exploratory factor analysis; IFSMT: Individual family self-management theory; ** If patients scored 4 or 5 on the Likert scale, they were considered high, and if they scored 1–3, they were considered low

In the EFA, the dimensions of some items from their original categorization were changed and 6 items did not load onto any one of the three factors (Table 3). Four of these 6 unloaded items were dropped from the subsequent validation process. However, the two unloaded items (PSMB2 and PSMB12) were retained because they were considered important based on their content.

#### Findings from qualitative discussion conducted after EFA

Discussions were held with the data collectors regarding each item before commencing the second round of data collection which was used for confirmatory factor analysis (CFA). Out of the six items that either did not load or had loadings below 0.3, four were omitted due to their limited importance to the questionnaire or redundancy, as they were covered by other items, resulting in a 28-item HIV-SM LMIC questionnaire. The remaining two items were retained for CFA due to their perceived significance. Additionally, one item (PSMB1) was revised to maintain its original concept, as data collectors had interpreted it differently. Further details and explanations can be found in Supplementary File A.

Another significant issue discussed with the data collectors was the inconsistency in patient responses across different items. Patients occasionally provided conflicting answers, often due to fear of judgment from providers or other personal reasons. For instance, they might initially assert strong adherence to medication but later disclose instances of missed doses. To mitigate this, we stressed the importance of data collectors capturing the genuine characteristics and realities of the patients.

### Self-management status of HIV patients

The responses to the 32-item HIV-SM LMIC questionnaire from study participants in the first and second rounds of the study are summarised in Table 3. All the 32 items have five response scales, and the first two categories (response 1 and 2) are categorised as low, while responses from 3 to 5 are categorised as high. In the first round of the study the responses of the study participants are high for each scale ranging from 78.9 to 99.6%. In the second round of the study the responses of the study participants are high for each item ranging from 75.7 to 99.7%. In the first round of the study, more than 90% of respondents responded high for 23 (72%) of the 32 items, and in the second round, more than 90% of respondents responded high for 24 (75%) items.

### Confirmatory factor analysis

Confirmatory factor analysis (CFA) was conducted using 28 items from the HIV-SM LMIC questionnaire. Five different types of CFA models (1 initial, 3 improved, and 1 final CFA model) were sequentially fitted to generate the final list of items for the HIV-SM LMIC questionnaire. Supplementary File B presents the initial fitted and improved CFA models. Table 4 outlines the final CFA model used to create the definitive HIV-SM LMIC questionnaire.

**Table 4:** Final confirmatory factor analysis result - item loadings and grouping

Dimensions	Items	Final CFA model - Coefficient (95% CI)		
Awareness and well being	CIF3	0.35 (0.22, 0.48)		
	CIF6	0.52 (0.40, 0.64)		
	PKB3	0.53 (0.42, 0.65)		
	PKB4	Dropped		
	PKB6	0.71 (0.59, 0.83)		
	PRN11	Dropped		
	PSF3	Dropped		
	PSR1	Dropped		
Self-regulations: emotional management and compliance to treatment plan.	CS3	0.67 (0.59, 0.75)		
	CS5	Dropped		
	CIF2	Dropped		
	PSR2	Dropped		
	PSR4	0.60 (0.52, 0.69)		
	PSR5	0.72 (0.64, 0.79)		
	PSR12	-0.56 (-0.65, -0.47)		
	PSR14	-0.37 (-0.48, -0.26)		
	PSR23	0.74 (0.67, 0.81)		
Self-management practices: Compliance to Self-care activities	PSMB1_01	0.65 (0.57, 0.72)		
	PSMB2	0.42 (0.32, 0.52)		
	PSMB7	0.18 (0.06, 0.30)		
	PSMB8	0.30 (0.19, 0.41)		
	PSMB12	Dropped		
	PSMB15	0.37 (0.26, 0.47)		
	PSMB19	0.63 (0.56, 0.71)		
	PSMB21	0.76 (0.70, 0.81)		
	PSMB22	0.70 (0.64, 0.77)		
	PSMB23	0.74 (0.68, 0.80)		
	PSMB24	0.76 (0.70, 0.81)		
covariance (e. PKB3, e. PSMB1_01)			0.35 (0.24, 0.47)	
covariance (e. PSR12, e. PSR14)			0.38 (0.28, 0.48)	
covariance (e. PSMB7, e. PSMB8)			0.37 (0.28, 0.47)	
covariance (e. PSMB7, e. PSMB15)			0.27 (0.16, 0.37)	
covariance (e. PSMB8, e. PSMB15)			0.34 (0.24, 0.44)	
covariance (SR_know, SR_skills)			-0.26 (-0.41, -0.11)	
covariance (SR_know, SM_practices)			0.32 (0.17, 0.46)	
covariance (SR_skills, SM_practices)			-0.61 (-0.71, -0.51)	
**Goodness of test of the model**				
LR test of model vs. saturated: chi2(162) (Prob > chi2 = 0.0000)			469.42	
Root mean squared error of approximation (RMSEA) with 90% CI			0.08 (0.07, 0.09)	
Akaike’s information criterion (AIC)			11785.39	
Bayesian information criterion (BIC)			12037.24	
Comparative fit index (CFI)			0.84	
Tucker-Lewis’s index (TLI)			0.81	
Standardized root mean squared residual (SRMR)			0.08	
Coefficient of determination (CD)			0.99	

The initial CFA model was constructed based on the results of the EFA, incorporating the three dimensions of the questionnaire. However, the model goodness of fit was not significant. Consequently, post-estimation statistics (i.e. Modification Indices (MI)), were employed to explore potential enhancements to the model. One notable observation was the potential misclassification of items across the three dimensions. Subsequently, four items displayed notably high MI values with their original EFA dimensions. These four items were then reclassified into new dimensions, after checking their alignment with the theoretical rationale of the respective dimensions. Table 5 describes reclassification of these items.

**Table 5:** Item reclassification based on the result of initial confirmatory factor analysis

Item code	EFA dimension	New (CFA) dimension	Remark
PSR14	Self-management practices	Self-regulations	Comply also with theoretical rationale.
PSMB1	Awareness and well being	Self-management practices	
PSMB2	Self-regulations	Self-management practices	
PSR12	Awareness and well being	Self-regulations	

As depicted in Table 4, the final CFA model resulted in the removal of 8 items to produce a 20-item HIV-SM LMIC questionnaire (4 items in awareness and wellbeing, 6 in self-regulation, and 10 in self-management practices dimensions). Despite having coefficients or loadings of less than 0.40, five items were retained in the final version of the questionnaire because of their theoretical importance. These items include CIF2 in awareness and wellbeing, PSR14 in self-regulation, and PSMB7, PSMB8, and PSMB15 in self-management practices dimension. Furthermore, seven items exhibited loading coefficients of 0.70 or higher, four items had coefficients between 0.60 and 0.69, the loading of three items fell within the range of 0.50 and 0.59, and one item had a loading of 0.42.

### HIV-SM LMIC final item characteristics with key patient attributes

**Fig. 3:**
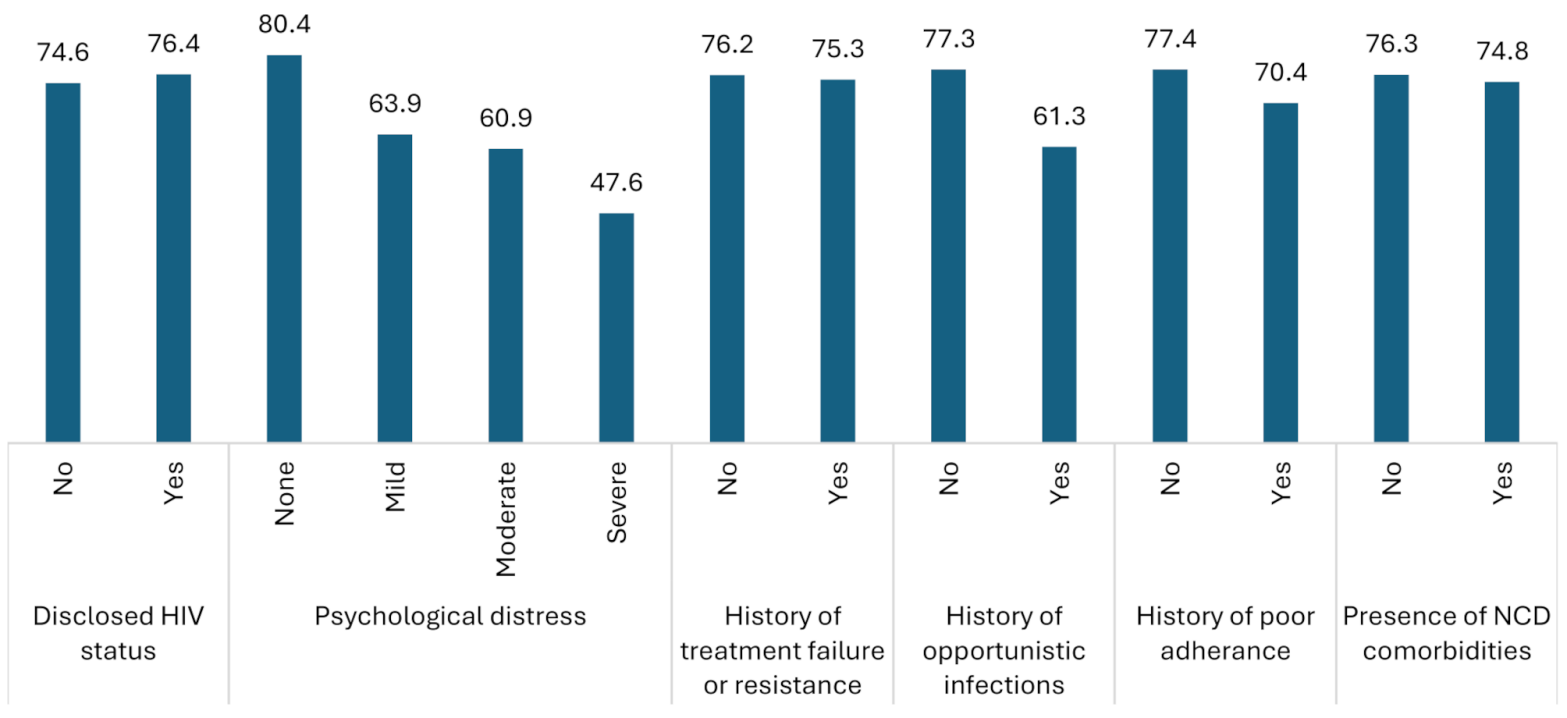
Mean of items across patient attributes

The 20 final items of HIV-SM LMIC questionnaire categorized into high and low in which the response and five categorized together as high the rest response categories as low. Some key patient characteristics which can measure treatment outcome of HIV patients were observed by means of the 20 items as shown in Fig. 3. There is no difference between mean of 20items among patient who disclosed and undisclosed their HIV status. The same is true also for occurrence of treatment failure and presence of NCD. However, there is significant difference of means of the items by psychological distress, occurrence of opportunistic infection and poor adherence history. Patients with psychological distress, opportunistic infection and history of poor adherence have low mean values than their counterparts.

Four items including CS3, PSR4, PSR5, and PSR23 have reverse score. Items include PSMB7, CS3_new, and PSMB8 have the lowest values as compared to other items, whereas PKB6, PSR12, and PSR14have the highest values. Table 6 shows the crosstabulation of the 20 final items with the key treatment outcome variables. More details of crosstabulation between patient characteristics and 20 items is described in supplementary file D.

**Table 6:** Key treatment outcomes crosstabulation with the 20 final items

Factor	Final items	Key characteristics of the patient													
		Disclosed HIV status		Psychological distress				Treatment failure or resistance		Opportunistic infections		Poor adherence		Presence of NCD	
		No	Yes	None	Mild	Moderate	Severe	No	Yes	No	Yes	No	Yes	No	Yes
Awareness and wellbeing	CIF3	84.0	82.9	85.4	78.2	60.0	57.1	81.5	93.3	83.4	78.6	83.5	81.0	82.7	85.7
	CIF6	91.4	83.5	86.4	80.7	70.0	71.4	85.0	82.7	84.8	83.3	86.6	76.0	83.9	90.0
	PKB3	72.8	76.7	79.1	69.7	50.0	42.9	74.1	89.3	77.3	61.9	77.0	72.0	76.2	75.7
	PKB6	85.2	94.0	94.4	87.4	90.0	85.7	92.4	94.7	92.9	90.5	92.4	94.0	92.5	94.3
Self-regulations: emotional management and compliance to treatment plan	CS3_new	55.6	54.6	59.8	41.2	30.0	14.3	53.1	65.3	56.6	31.0	53.8	59.0	54.6	55.7
	PSR4_new	63.0	73.3	75.3	61.3	50.0	71.4	71.8	72.0	73.0	57.1	73.1	66.0	72.1	70.0
	PSR5_new	69.1	81.0	84.9	63.9	40.0	57.1	80.2	73.3	80.9	59.5	81.3	70.0	79.6	77.1
	PSR12	92.6	93.8	96.7	84.9	90.0	57.1	94.0	90.7	93.8	90.5	95.2	86.0	93.5	94.3
	PSR14	96.3	97.9	98.6	95.0	100.0	85.7	97.9	96.0	98.1	92.9	98.5	94.0	97.6	98.6
	PSR23_new	64.2	79.6	81.9	65.5	60.0	28.6	79.0	66.7	78.6	61.9	80.7	62.0	76.4	84.3
Self-management practices: Compliance to Self-care activities	PSMB1_01	85.4	79.2	87.3	55.2	88.9	66.7	80.8	70.8	81.9	62.1	81.7	72.2	83.1	59.0
	PSMB2	58.0	68.8	72.5	52.9	50.0	14.3	68.1	61.3	69.6	38.1	68.1	63.0	67.6	64.3
	PSMB7	46.9	44.4	48.5	35.3	20.0	14.3	45.5	40.0	46.4	23.8	46.2	38.0	44.0	50.0
	PSMB8	56.8	55.0	60.7	40.3	20.0	28.6	54.3	61.3	56.3	42.9	54.4	59.0	55.4	54.3
	PSMB15	70.4	64.8	70.4	51.3	50.0	42.9	65.6	65.3	66.7	52.4	66.6	61.0	66.2	61.4
	PSMB19	85.2	75.6	80.7	67.2	60.0	42.9	76.3	81.3	78.8	54.8	77.7	74.0	77.4	74.3
	PSMB21	87.7	86.0	90.6	73.1	90.0	42.9	86.2	86.7	87.7	69.0	87.6	80.0	87.2	80.0
	PSMB22	77.8	76.3	84.0	51.3	80.0	42.9	77.6	69.3	77.8	59.5	78.7	66.0	77.4	70.0
	PSMB23	69.1	78.1	82.6	58.8	70.0	42.9	78.0	69.3	78.2	59.5	80.3	61.0	76.8	77.1
	PSMB24	80.2	82.3	88.0	65.5	50.0	42.9	82.9	76.0	84.0	57.1	83.7	74.0	82.3	80.0

## Discussion

The 32-item HIV-SM LMIC questionnaire underwent further validation for content and construct validity through two rounds of data collection involving 561 HIV patients. During this process, 12 items were dropped (4 during the exploratory factor analysis [EFA] and 8 during the confirmatory factor analysis [CFA]), four items were reclassified from the EFA suggested dimension classification to other dimensions based on the CFA results, and one item was revised. This resulted in a 20-item HIV-SM LMIC questionnaire with three dimensions: awareness and wellbeing, which has 4 items; self-regulation (i.e. Emotional management and compliance with the treatment plan), which has 6 items; and self-management practices, which has 10 items. The final CFA model demonstrated an acceptable level of goodness of fit.

The ultimate three dimensions in our HIV-SM LMIC questionnaire and the IFSMT model, which formed the basis for our studies, differ in several aspects [45]. The first discrepancy is in the number of dimensions: the IFSMT model has four dimensions (context, process, proximal outcome and distal outcome), whereas the HIV-SM LMIC questionnaire has three dimensions. The second discrepancy relates to the shift and merging of some dimensions in the new questionnaire that differ from the original IFSMT model. For example, the awareness and well-being dimension consists of items originally developed in the context and process dimensions. The reasons for this discrepancy may be that the IFSMT model has not been tested, does not reflect current practice, which is in contrary with the new HIV-SM LMIC dimensions, and was developed based on developed country contexts. These findings highlight the need to revise the IFSMT to better reflect the realities faced by HIV patients, families, and service providers in developing countries. The current study can contribute significantly to this regard, especially for the contexts of developing countries.

The decision to drop or retain items was based both on the results of statistical analysis (EFA and CFA) and theoretical rationales, as usually recommended [51–53]. Some items with low factor loading were kept in the final 20-item HIV-SM LMIC questionnaire due to their theoretical importance. Among the 12 items that were dropped, some items had high factor loading but were excluded due to overlapping concepts, or lack of significance to the context. Relying solely on statistical results or theoretical assumptions can result in an inaccurate and unreliable questionnaire. Psychometric tools measure personality traits indirectly through responses to a range of items [43], which can introduce various errors, including measurement errors and lack of sensitivity to population variations, affecting the accuracy and reliability of the items [52]. In producing the final 20-item questionnaire, greater emphasis was placed on theoretical rationales for keeping the items rather than making decisions purely based on statistical results.

The inclusion of various key areas of self-management practices is essential, as revealed by the opinions of experts, service providers, and patients themselves [23, 24]. This helps to address the multifaceted needs related to the chronic condition and improve their quality of life [16, 37]. Thus, self-management questionnaires should be comprehensive, assessing all key areas and capturing context-specific conditions [23, 36]. The current 20-item HIV-SM LMIC questionnaire with three dimensions is comprehensive and covers key areas of self-management. The “awareness and wellbeing” dimension includes items related to dedicating time for treatment, the importance of sleep and physical activity, and a clear understanding of disease-related issues. The “self-regulation” dimension comprises items related to resilience to stigma, mental distress, emotional problems, daily routines, and treatment adherence. The third dimension includes items related to self-management activities. These dimensions encompass items that should be used to assess self-management as mentioned by various authors [39–41, 54–56].

When compared to other self-management questionnaires developed elsewhere [39, 40, 57], the HIV-SM LMIC questionnaire is within the context of developing countries to assess various dimensions of self-management. Questionnaires developed in the context of developed countries often do not reflect the contexts of developing countries, whereas the HIV-SM LMIC questionnaire is based on a comprehensive theoretical framework and tested in the contexts of developing countries. However, before implementing the questionnaire in other low- and middle-income countries, it remains important to conduct cross-cultural validation. Different cultures, social norms, and practices influence how questions are understood and how responses vary [58]. Therefore, it is crucial to test the items across various cultures to increase the relevance, comparability, and reliability of the HIV-SM LMIC questionnaire [59, 60].

The 20-item HIV-SM LMIC questionnaire, specifically designed for chronic HIV patients, is unique in its ability to assess the complex nature of self-management among HIV patients in low- and middle-income countries. Some items that characterize specific groups of HIV patients, such as newly diagnosed patients who behave differently than chronic HIV patients, were dropped during the analysis. Therefore, the HIV-SM LMIC questionnaire should be customized differently for newly diagnosed HIV patients. In addition, almost three-quarters of the items were rated highly by more than 90% of respondents, indicating homogeneity of patients in key characteristics. For instance, 98% of HIV patients have an undetectable viral load, and 93% are on a first-line ART regimen. This is mainly because respondents were recruited from outpatient ART clinics, where patients come to refill their ART drugs—these patients are not sick or experiencing poor treatment outcomes. Future validation of the HIV-SM LMIC questionnaire should therefore include patients with current opportunistic infections, those on second or higher ART regimens, or those admitted to the hospital.

This study does not present the scoring method or cut-off points for the HIV-SM LMIC questionnaire, as multiple validation studies are needed to demonstrate the association between the self-management item scores and key HIV treatment outcomes, such as quality of life, adherence, and the occurrence of opportunistic infections. Determining cut-off points is part of predictive validity, which should be established after validating the questionnaire in different settings, and contexts [61–63]. Validation of the HIV-SM LMIC questionnaire with measures of HIV treatment outcomes, such as quality of life, adherence, and incidence of opportunistic infections in different settings, is critical.

### Strength and limitation of the study

It is known that development and validation of new questionnaires should adhere to an iterative process guided by robust scientific methodologies. In this study, the authors adopted a phased approach and rigorous scientific methods to develop and validate the HIV-SM LMIC questionnaire. Moreover, the sample recruited for the validation process in two rounds was optimal, encompassing a diverse group of patients from various levels of health facilities, including health centers (primary level), general hospitals (secondary level), and specialized referral hospitals (tertiary level). However, the study is not without limitations. Social desirability bias and a sense of urgency during interviews with HIV patients might have influenced the responses to items rated high. To mitigate these limitations, data collectors provided detailed explanations about the aims of the study to the participants. The authors also closely monitored the data collection process daily to ensure data quality, utilizing data quality queries. Whenever issues arose, the authors communicated with the data collectors daily to promptly resolve them.

## Conclusion and recommendation

The 32-item HIV-SM LMIC questionnaire underwent both an exploratory factor analysis (EFA) phase and a confirmatory factor analysis (CFA) phase, resulting in a refined 20-item HIV-SM LMIC questionnaire. The decision to drop 12 items during the validation process was based on their factor loadings and concrete rationales. Some items shifted from their original dimensions to other dimensions, underscoring the need for a revision of the IFSMT model to better align with the actual realities faced by patients, families, and providers. Consequently, the authors recommend further testing and validation of the IFSMT model. Moreover, the HIV-SM LMIC questionnaire should be validated in other low- and middle-income countries to ensure cross-cultural and predictive validity. Since the current iteration of the questionnaire assesses the self-management practices of chronic HIV patients, it should be adapted for newly diagnosed HIV patients as well. Moreover, it should be validated among HIV patients with poor treatment outcomes, who are admitted or have virological failure or have opportunistic infections.

## Electronic supplementary material

Below is the link to the electronic supplementary material.

Supplementary Material 1

Supplementary Material 2

Supplementary Material 3

Supplementary Material 4

## Data Availability

Data will be provided by the corresponding author (tege2004@gmail.com) upon reasonable request.
